# Synthesis and characterization of poly(styrene-co-divinylbenzene) and nanomagnetite structures

**DOI:** 10.1016/j.mex.2022.101764

**Published:** 2022-06-19

**Authors:** Mariana de Oliveira Reis, Ricardo Geraldo de Sousa, Adriana de Souza Medeiros Batista

**Affiliations:** a Department of Nuclear Engineering, Universidade Federal de Minas Gerais, Belo Horizonte, Brazil; b Department of Chemical Engineering, Universidade Federal de Minas Gerais, Belo Horizonte, Brazil; c Department of Anatomy and Image, Universidade Federal de Minas Gerais, Belo Horizonte, Brazil

**Keywords:** Composite materials, Magnetic polymers, Adsorbent materials

## Abstract

The use of iron oxide nanoparticles for the synthesis of adsorbents materials with application in contaminated water remediation is based on both their magnetic and adsorption properties. Poly(styrene-co-divinylbenzene) - Poly(Sty-co-DVB)microparticles are widely known for this use and the addition of magnetite comes to add magnetic properties, however it should not limit the functionalization processes for formation of cationic and/or anionic resins. Thus, there is a concern about the stability of magnetite in acidic environments, as required by the sulfonation process, for example. Furthermore, the synthesis of composites with magnetic nanoparticles is a challenge due to the strong magnetic dipole interactions between particles resulting from the high specific surface area, making them susceptible to aggregation. As well as oxidation during the synthesis process can compromise the structure of the initial magnetic material. In this sense, the present work validated processes for the synthesis of Poly(Sty-co-DVB)/magnetite microparticles that sought, as a final result, the incorporation of magnetite aiming to preserve its initial structural conformation. Thus, started from original synthesis method aimed at the production of Poly(Sty-co-DVB) microparticles without magnetite, to methods in which magnetite nanoparticles is added through two distinct processes, different regarding the use or not of oleic acid in its preparation.

In this method article we show:

• Combination of methods to preserve the structure of magnetite in the Poly(Sty-co-DVB)/magnetite composite.
• Simple method of adding magnetite to the Poly(Sty-co-DVB)/magnetite composite synthesis process.
• Magnetite treatment method with oleic acid in Poly(Sty-co-DVB)/magnetite composite synthesis.

The samples were characterized using Fourier-transform infrared spectroscopy (FTIR), thermogravimetric analysis (TGA) and Mössbauer spectroscopy to evaluate the chemical modifications that were introduced in the original synthesis method.

## Graphical abstract

**Figure:**
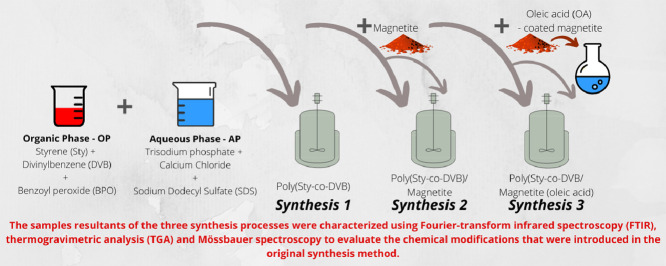

Specifications table

**Table:** 

Subject area:	Materials Science
More specific subject area:	Polymeric microparticles
Method name:	Synthesis of the poly(styrene-co-divinylbenzene)/magnetite microparticles by suspension polymerization.
Name and reference of original method:	Synthesis of the poly(styrene-co-divinylbenzene) microparticles by suspension polymerization.
	Rodrigo, Raúl, Claudio A. Toro, and Jorge Cuellar. "Morphological characteristics of poly (styrene-co-divinylbenzene) microparticles synthesized by suspension polymerization." Powder technology 247 (2013): 279-288.
Resource availability;	Iron oxide source - Sigma-Aldrich®: nanopowder, 50-100 nm particle size (SEM), 97% trace metals basis. https://www.sigmaaldrich.com/BR/pt/product/aldrich/637106?context=product

## Method details

The adsorption process is widely studied for the remediation of wastewater, being a viable alternative for trace concentrations of contaminants. In this sense, the use of nanoparticulate iron oxide in the synthesis of polymeric adsorbents has the advantage of associating magnetic and adsorptive properties [1], [2], [3], [4]. Styrene-divinylbenzene copolymer microparticles (Poly(Sty-co-DVB)) are widely known for this use, and the addition of magnetite to the synthesis process aiming to add magnetic properties must be done without limiting the adsorbent functionalization processes, with the purpose of forming cationic and/or anionic resins [5]. Knowing that the structure of the initial magnetic material can be compromised by oxidation during the synthesis process, and that the aggregation of nanoparticles is a challenge to be faced in this process [6]. The study in this work was conducted in order to explore these aspects.

Thus, the present work validated processes for the synthesis of Poly(Sty-co-DVB)/magnetite microparticles that sought, as a final result, the incorporation of magnetite in order to preserve its initial structural conformation. Started from the original synthesis method [7] aimed at the production of Poly(Sty-co-DVB) microparticles without magnetite, to methods in which magnetite nanoparticles are added through two distinct processes, regarding the use or not of oleic acid in their preparation.

Synthesis of Poly(Sty-co-DVB) microparticles with and without magnetite

For the synthesis of poly(styrene-co-divinylbenzene) microparticles in the polymerization reactor, the following reagents were used:

• Sigma-Aldrich® Styrene (Sty): ≥99%.
• Divinylbenzene (DVB) Aldrich® Chemistry: 55%.
• Benzoyl peroxide (BPO) Vetec: solid; anhydrous min. 65%, water max. 25%.
• Aldrich® Chemistry Trisodium phosphate: 96%.
• Sigma-Aldrich® calcium chloride: anhydrous, granular, ≤7.0 mm; ≥93%.
• Sigma-Aldrich® Sodium Dodecyl Sulfate (SDS): ≥99%.

The monomers are used for formation of the organic phase (OP) after washed with a 10% aqueous NaOH solution to remove the inhibitor, 4-tert-butylcatechol, and then with deionized water until neutralization. The initiator, benzoyl peroxide (BPO), was used as received. The last three reagents cited (Na_3_PO_4_, CaCl_2_ and SDS) were used for the formation of the aqueous phase (AP). The proportion of reagents for the formation of OP is 4% of DVB, by volume, of Sty used. Thus, aiming at the total volume of the reaction medium at 150 mL, with the ratio in volume of OP to AP of approximately 1:4, 30 mL is calculated for Sty+DVB+BPO and 120 mL of Na_3_PO_4_+CaCl_2_+SDS.

The procedure begins by removing an aliquot of each monomer and transferring it to a decanting funnel to wash both the Sty and the DVB to remove the stabilizer, the inhibitor 4-tert-butylcatechol. Considering possible losses in the washing procedure, 36 mL of Sty and 1.44 mL of DVB were used (in practice, 2.62 mL of DVB solution 55% was pipetted to wash the monomer, in order to obtain that DVB volume). Washing is done with 10% NaOH (m/v), using a 1:1 volume ratio for each reagent placed in a decanting funnel, separately, shaking and removing the denser phase afterwards, which is done in triplicate. Afterwards, washing with distilled water is continued, which is also done three times, in a similar procedure; however, twice the volume of each reagent is used per wash, i.e., the volume ratio is 2:1 water to monomers. Finally, the washed monomers are collected.

The reagents Na_3_PO_4_ and CaCl_2_ were used to obtain the precipitate Ca_3_(PO_4_)_2_, in 1.43% (m/v) of the AP. For this, 1.85 g of CaCl_2_ and 1.82 g of Na_3_PO_4_ were weighed to be used. For the BPO initiator and the SDS surfactant, the amounts used of each followed the proportion of 4% (m/v) of OP and 0.0167% (m/v) of AP, respectively. Therefore, 1.50 g of BPO and 0.02 g of SDS were used. The solids that will form the aqueous phase (Na_3_PO_4_ and CaCl_2_) were solubilized in distilled water separately and then joined to process the precipitation reaction, with the SDS being added at that time. With the AP prepared and before adding the OP to the reactor, the monomers were added with BPO. Thus, in possession of the phases prepared separately, OP and AP are added to the reactor vessel to initiate the polymerization in automated reactor OptiMax™ 1001 Thermostat, from Mettler Toledo.

The reactor was programmed for the following sequence of operations:

• Step 1: 900 rpm of agitation, at room temperature (25 °C), for 3 h;
• Step 2: Maintenance of 900 rpm of medium agitation speed, changing the temperature to 82 °C;
• Step 3: Maintenance of the reaction medium temperature of 82 °C, modifying the medium agitation speed to 500 rpm, for 5 h.

After polymerization, the reactor is cooling at room temperature (25 °C) and turned off. The polymerization product is removed and vacuum filtered, and washed with hot water. The remaining material is placed in contact with a 2 N hydrochloric acid solution under stirring for 1 h, and then filtered. The solid obtained is then placed in contact with absolute ethanol for another 2 h, under stirring, filtered again under vacuum and the solid obtained is now placed in an oven to dry at 40 °C for 24 h.

For the synthesis of poly(styrene-co-divinylbenzene)/magnetite microparticles, 3.3 g (10% m/m of the organic phase) of magnetite nanoparticles (Sigma-Aldrich®: 97%, 50–100 nm) were used initially directly into the reaction medium, only dispersing the particles in an ultrasonic bath for 3 min. Thus, the polymerization proceeded as described for the synthesis without magnetite.

In the other hand, the poly(styrene-co-divinylbenzene)/magnetite microparticles were synthesized too with a previous preparation of magnetite, using oleic acid (Synth®). Thus, 150 mL of water were placed in a round-bottom flask under a heating mantle, which was adjusted to 65 °C to process the experiment. After adding 3,3 g magnetite, 50 mL oleic acid were gradually added. Stirring of the medium was maintained for 30 min. At the end of the process, to aid in the removal of the aqueous phase, absolute ethanol was mixed with the medium, awaited decantation and subsequent separation with filtration of the excess water. Before being used in polymerization, the sample was allowed at room temperature until remove excess water.

**Fig. 1:**
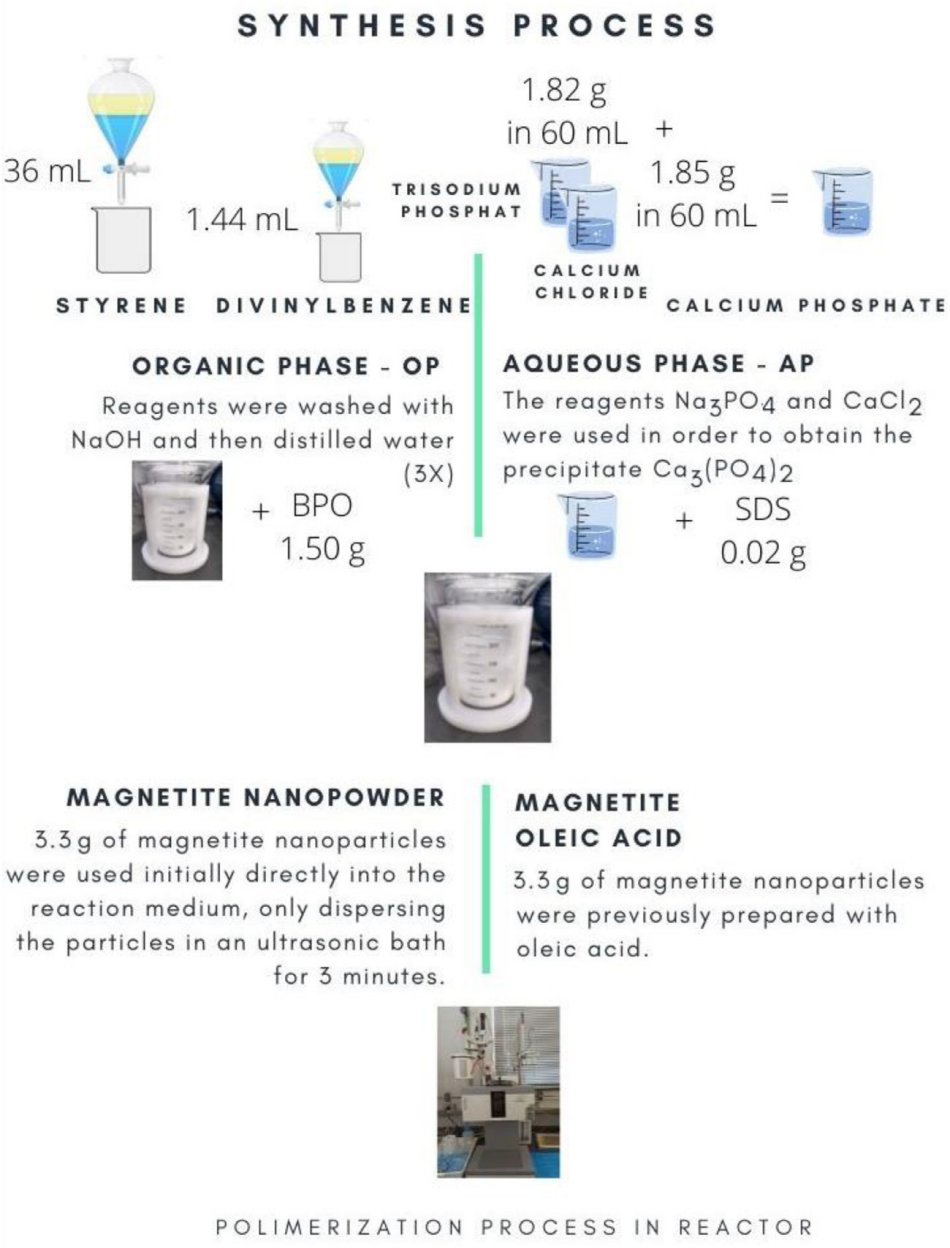
Reagents preparation scheme for Poly(Sty-co-DVB)/Magnetite microparticles synthesis.

In Fig. 1 present an illustrative scheme of the steps that precede the polymerization process.

**Fig. 2:**
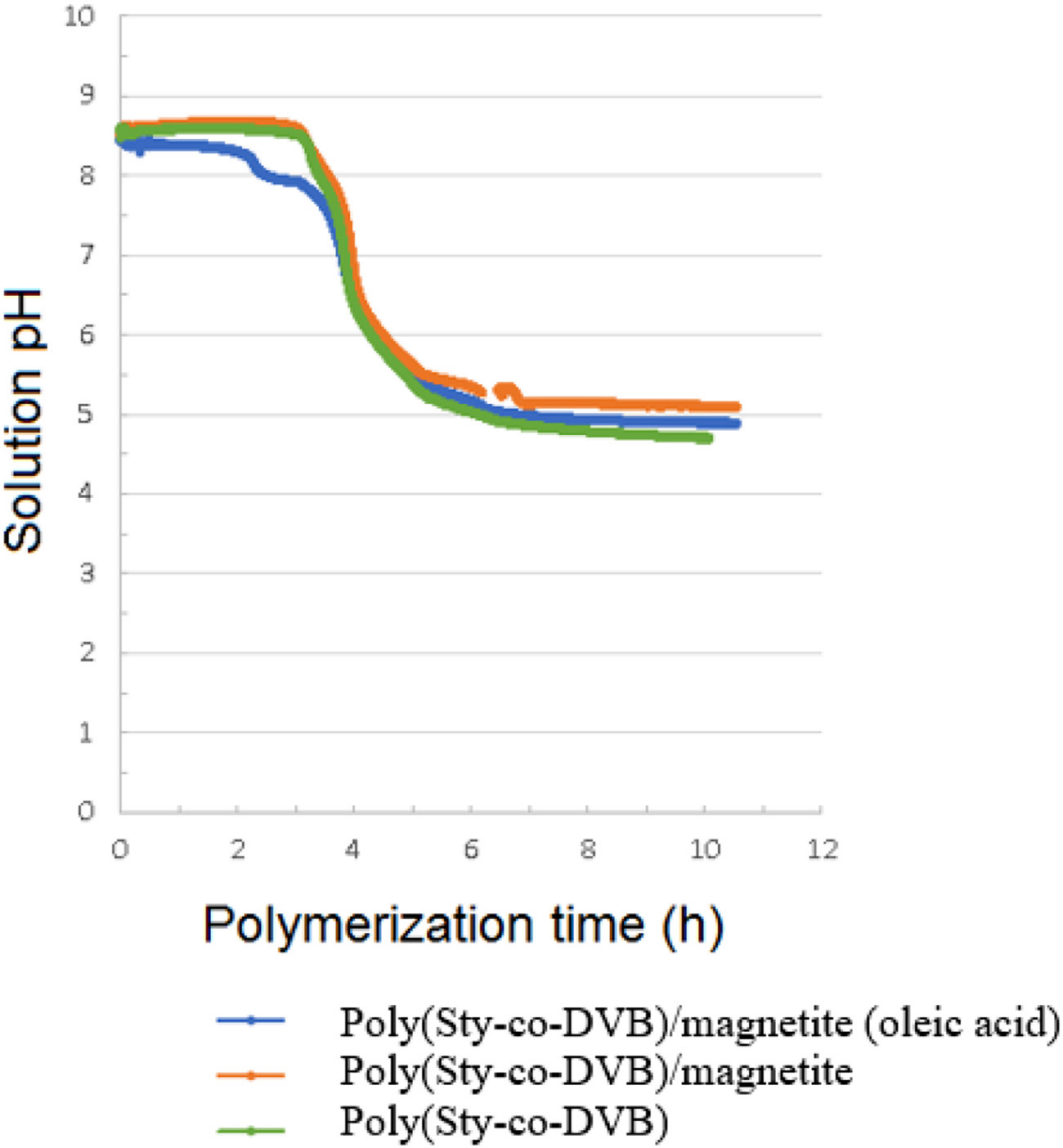
Solution pH variation by polymerization time.

As can be seen in Fig. 2, the pH of the medium undergoes great variation during the synthesis process, going from basic to acid (from approximately 8,5 to close to 5 at the end of the experiment), for the different polymerizations carried, with or without the addition of magnetite. This inversion occurred during in the step 2, when the system temperature increased from 25 °C to 82 °C, with stirring at 900 rpm. This stage, which took about 120 min to complete, corresponds to the period in which the polymerization reaction to be starts. The initial period of 3 h under stirring at 900 rpm (Step 1) was used, as described by Rodrigo; Toro; Cuellar (2013) [7], in order to ensure that the average size of the monomer droplets would reach a constant value.

In Fig. 2 can see that the moment of change in pH occurred between 3 h and 5 h during the synthesis process. Previous works indicate that the variation in the pH of the medium may be related to the decomposition of the initiating agent (BPO), which produces CO_2_, reducing the pH of the medium. At pH 5, tricalcium phosphate (Ca_3_(PO_4_)_2_) can dissociate and dissolve in the reaction medium, decreasing its ability to prevent coalescence of monomeric droplets / polymeric granules, since this impediment can only be achieved with tricalcium phosphate is in the form of powder adhered to the surfaces of these droplets/beads and not dissolved in solution. In order to mitigate this problem, tests using ammonium hydroxide (NH_4_OH) were carried out in another works, aiming to maintain the basic pH in the reaction medium. The factors that affect the coalescence/agglomeration of microparticles during the polymerization process of poly(styrene-co-divinylbenzene) were investigated, indicating that a high surfactant concentration value (on the order of 0.01% (m/v) in relation to aqueous phase) favors the formation of microparticles with a narrower size distribution. However, the interaction analysis of the factors (ammonium hydroxide and surfactant concentration) points to a negative effect for the use of the same level for both [6]. Thus, in the present work, chose to carried out the polymerization reaction without the use of ammonium hydroxide and with the surfactant at a concentration slightly higher than 0.01% (m/v) of the aqueous phase. In addition, the polymerization was carried out by increasing the temperature by means of a heating ramp, with a heating rate lower than that indicated in the reference article [7], in order to promote a longer and more controlled reaction to obtain more similar morphologically particles.

Mössbauer spectroscopy was performed in a conventional spectrometer, equipped with a transducer controlled by a command unit by linear function, and proportional counter type radiation detectors, with a gas chamber with 97% of Krypton and 3% of CO_2_, at the pressure of 1 atm. Measurements were made using transmission geometry, constant acceleration and ^57^Co source kept at room temperature. Experimental data were fitted by Lorentzian functions using least squares using the NORMOS 90 program (R.A. Brand, Laboratorium für Angewandte Physik, Universität Duisburg, D-47048, Duisburg-Germany). FTIR spectra were obtained incorporating the materials to KBr pellets on a Thermo Scientific equipment model Nicolet 6700, with 128 scans ranging from 4000 to 400 cm^−1^ at a resolution of 4 cm^−1^. Thermogravimetric analysis (TGA) was conducted on a TA Instruments SDT Q600 analyzer, subjected to a heating rate of 10 °C min^−1^ under an atmosphere of nitrogen flowing at 100 mL min^−1^; the sample mass was between 5.0 and 5.5 mg for all tests and the temperature ranged from 30 °C to 600 °C.

## Method validation

**Fig. 3:**
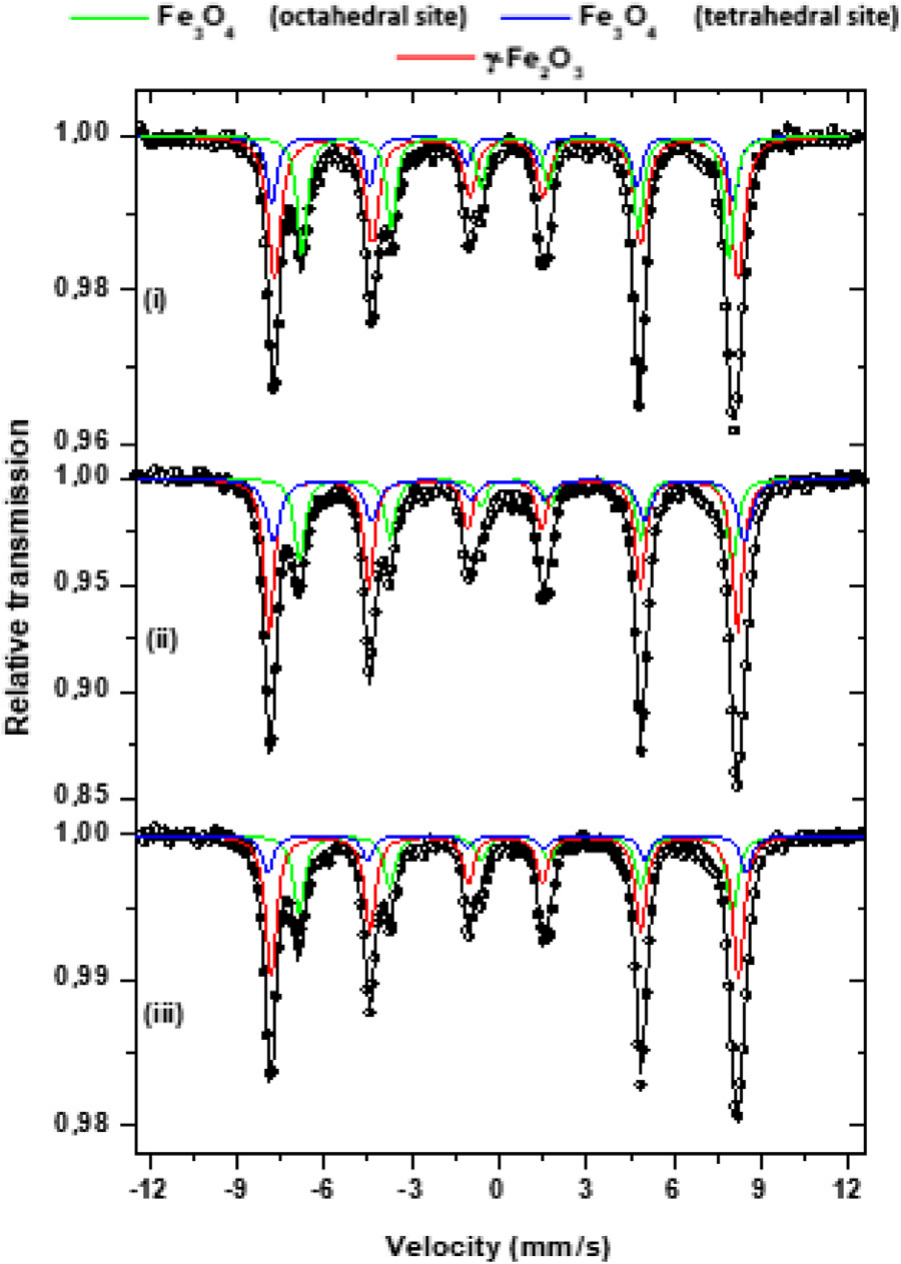
^57^Fe Mössbauer spectra obtained at room temperature for three samples: (i) magnetite nanoparticles - Sigma-Aldrich®, (ii) Poly(Sty-co-DVB)/magnetite microparticles and (iii) Poly(Sty-co-DVB)/magnetite microparticles treated with oleic acid.

The Mössbauer spectra were obtained at room temperature and fitted with three sets of sextets related parameters typical of iron oxides, magnetite (Fe_3_O_4_) and maghemite (γ-Fe_2_O_3_), as shown in Fig. 3.

The sextets identified in Fig. 3 by the blue lines refer to the tetrahedral Fe^3+^ sites, and those identified by the green lines to the octahedral (Fe^2+^, Fe^3+^) sites of the magnetite; the red line was associated with maghemite. The adjustment of hyperfine parameters obtained by Mössbauer spectra are shown in Table 1. As can be seen by spectra and Table 1, the relative intensities of the valleys for maghemite are slightly larger for the composites than for the pure nanoparticulate magnetite sample, resulting in a larger relative area as well. This difference is more significant between the pure sample and the composite sample in the absence of oleic acid. In stoichiometric magnetite, the ratio of Fe^3+^ and Fe^2+^ ions is 2:1, and under some conditions the magnetite phase can oxidizes into the maghemite phase with the conversion of Fe^2+^ ions into Fe^3+^ ions [8]. Considering the relative area obtained (Table 1), it can be noted that the ratio between the octahedral and tetrahedral sites for Poly(Sty-co-DVB)/magnetite and Poly(Sty-co-DVB)/magnetite microparticles treated with oleic acid samples, 1,93 and 1,72, respectively, are close to the ratio of the magnetite nanopowder (Sigma-Aldrich®) sample (1,85). The ratio between the octahedral and tetrahedral sites for all samples are close to the stoichiometric ratio 2:1.

**Table 1:** Hyperfine parameters obtained at room temperature of the samples: (i) magnetite nanoparticles - Sigma-Aldrich®, (ii) Poly(Sty-co-DVB)/magnetite microparticles and (iii) Poly(Sty-co-DVB)/magnetite microparticles treated with oleic acid.

Sample	Compound/oxidation state		δ (mm/s) ± (0,05 mm/s)	Δ/ε (mm/s) ± (0,05 mm/s)	B_HF_ (T) ± (0,5 T)	Relative area (%) ± (1)
i	Fe_3_O_4_ γ- Fe_2_O_3_	Octahedral Tetrahedral	0.64 0.27 0.30	0.03 -0.05 0.06	45.8 49.0 49.8	37 20 43
ii	Fe_3_O_4_ γ- Fe_2_O_3_	Octahedral Tetrahedra	0.66 0.24 0.33	0.03 -0.03 0.01	46.1 49.6 50.1	27 14 58
iii	Fe_3_O_4_ γ- Fe_2_O_3_	Octahedral Tetrahedral	0.65 0.26 0.31	0.02 -0.03 0.01	46.1 49.7 50.0	31 18 51

**Fig. 4:**
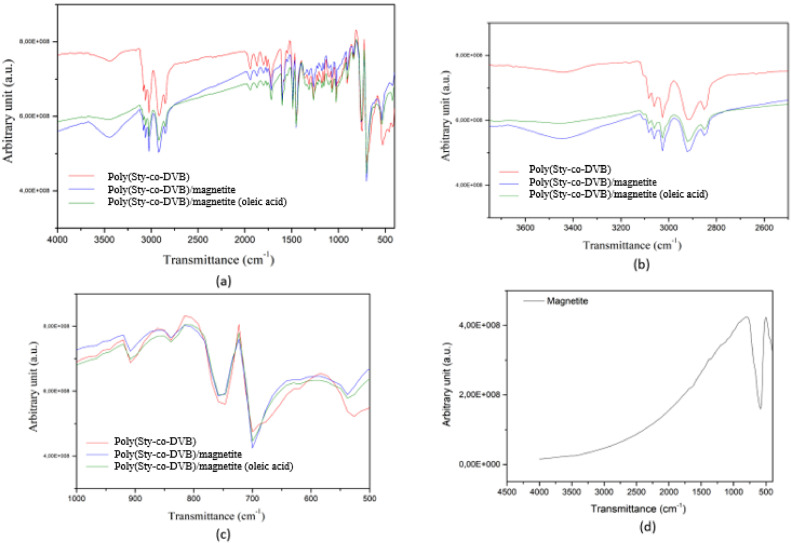
FTIR spectra of Poly(Sty-co-DVB), Poly(Sty-co-DVB)/magnetite and Poly(Sty-co-DVB)/magnetite treated with oleic acid microparticles for regions (a) 4000 – 400 cm^−1^, (b) 3750 - 2500 cm^−1^ and (c) 1000 - 500 cm^−1^, and (d) spectrum for magnetite only.

The Poly(Sty-co-DVB) samples with and without magnetite and oleic acid and pure magnetite sample were analyzed by FTIR and the spectra are shown in the Fig. 4, for three regions: (a) and (d) 4000 – 400 cm^−1^, (b) 3750 - 2500 cm^−1^ and (c) 1000 - 500 cm^−1^. Some absorption bands present in these FTIR spectra and their attributions are shown in Table 2. It is possible to observe bands in all FTIR spectra (Fig. 4(a)) for samples in the absorption region of 3100 to 3000 cm^−1^, related to the stretching of the Csp^2^-H bond of aromatic rings, between 3000 and 2800 cm^−1^ related to Csp^3^-H stretch (these absorption bands can also be seen in more detail in the Fig. 4(b)), and between 1500 and 1400 cm^−1^, due the angular deformations from CH bonds in the plane. These absorption bands are characteristic for Poly(Sty-co-DVB) copolymer [5,9].

**Table 2:** Absorption bands highlighted in FTIR spectra for all samples.

Bond	Positions in the spectrum obtained (cm^−1^)		
	Poly(Sty-co-DVB)	Poly(Sty-co-DVB)/ magnetite	Poly(Sty-co-DVB)/ magnetite (oleic acid)
Hydroxyl groups	3442	3442	3442
Csp^2^-H stretch	3084, 3060, 3026	3084, 3060, 3026	3084, 3060, 3026
Csp^3^-H stretch	2922, 2852	2922, 2852	2922, 2852
CH deformation in the plane	1487, 1452	1487, 1452	1487, 1452
C-H flexural vibrations outside the plan of the benzene ring	746, 700, 526	758, 700, 538	758, 700, 538
Fe-O		700, 538 585 (for the FTIR spectrum of magnetite only)	700, 538 585 (for the FTIR spectrum of magnetite only)

In the absorption region between 3600-3300 cm^−1^ (attributed to hydroxyl groups) [10] was observed a large band in all FTIR spectra (Fig. 4(b)). The presence this band may be due to the presence of traces of solvents (washing water and/or absolute ethanol used to neutralize the product obtained in the synthesis) in the samples.

The peaks in FTIR spectra at 758, 746, 700, 538 and 526 cm^−1^ (Fig. 4 (c)) for the three samples can be represent benzene disubstituted ring of the Poly(Sty-co-DVB), present in all samples [5,11,12].

The FTIR spectrum shown in the Fig. 4(d) present only an absorption band at 585 cm^−1^, that can be associated with magnetite nanoparticles (Fe-O bonds) [9].

It can be noted that peaks in FTIR spectra at 700 cm^−1^ (Fig. 4 (c)) and between 550-500 cm^−1^, for the three samples, presents small differences. These peaks are near of absorption bands attributed to Fe-O bonds (670 cm^−1^) [5] and (580 cm^−1^) [9], respectively, present in the samples with magnetite. The low amount of magnetite added to the polymer may justify the small differences and displacement in these absorption bands between the FTIR spectra these four samples (Fig. 4 (c) and (d)).

**Fig. 5:**
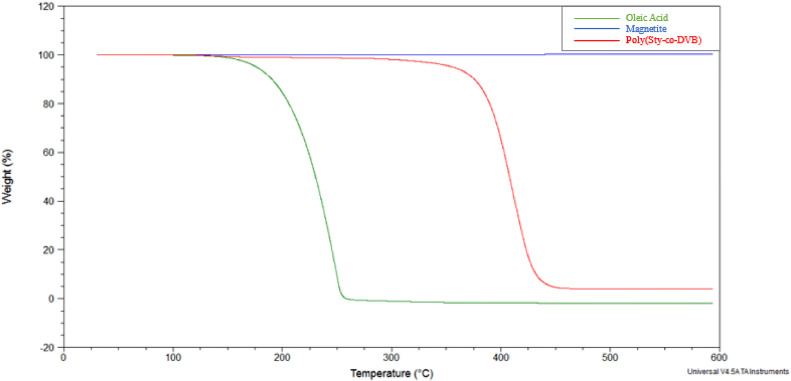
TGA curves for Poly(Sty-co-DVB) microparticles, magnetite nanoparticles and oleic acid samples.

**Fig. 6:**
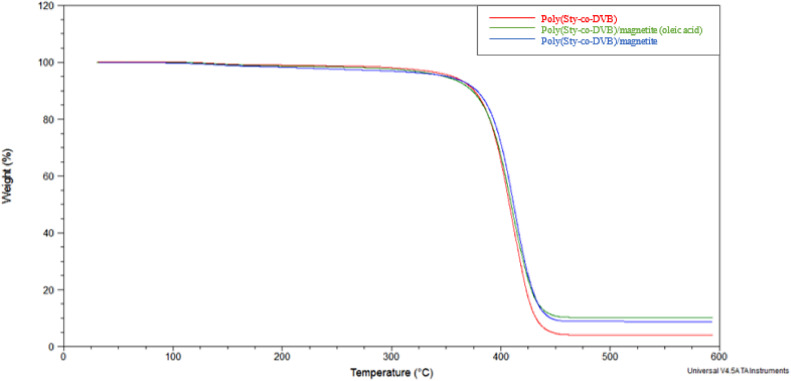
TGA curves for Poly(Sty-co-DVB), Poly(Sty-co-DVB)/Magnetite and Poly(Sty-co-DVB)/Magnetite/Oleic Acid samples.

The TGA curves for the Poly(Sty-co-DVB) microparticles, magnetite nanoparticles and oleic acid samples, shown in Fig. 5, were very well defined. In Fig. 6, the TGA curves obtained for the samples Poly(Sty-co-DVB), Poly(Sty-co-DVB)/Magnetite and Poly(Sty-co-DVB)/Magnetite/Oleic Acid microparticles presented similar profiles, that is, two mass losses. From the equipment program software, all TGA curves shown in Figs. 5 and 6 and their derivatives were analyzed and the results related to the mass losses, in two temperature intervals, and the degradation maximum temperatures (T_deg.max._) of these losses are presented in Table 3. The Tdeg.max. is the temperature where the decomposition rate of the thermal event reaches its maximum level, being obtained by the TGA curve derivate.

**Table 3:** Results of mass loss and degradation maximum temperatures for Poly(Sty-co-DVB) microparticles, magnetite nanoparticles, oleic acid, Poly(Sty-co-DVB)/Magnetite and Poly(Sty-co-DVB)/Magnetite/Oleic Acid microparticles samples.

SAMPLE	MASS LOSS (%)			T_deg. max._ (°C)		
	30 °C - 300 °C	300 °C - 600 °C	Residue		30 °C - 300 °C	300 °C - 600 °C
Poly(Sty-co-DVB)	1,9	94,0	4,1		137	409
Magnetite	—	—	100,0		—	—
Oleic Acid	100,0	—	0,0		247	—
Poly(Sty-co-DVB)/Magnetite	3,1	88,2	8,7		135	413
Poly(Sty-co-DVB)/Magnetite/Oleic Acid	2,4	87,5	10,1		133	410

In the temperature range investigated, 30 °C to 600 °C, magnetite showed no mass loss, while oleic acid degraded completely between 30 °C and 300 °C, with the degradation maximum temperature of the 247 °C. In the TGA curve for the Poly(Sty-co-DVB) copolymer, two consecutive mass losses were observed: 1.9% between 30 °C and 300 °C, with a Tdeg.max. of 137 °C, and 94.0% between 300 °C and 600 °C, with a Tdeg.max. of 409 °C. The first loss may be due to the presence of traces of solvents (washing water and/or hydrochloric acid and absolute ethanol used to neutralize the product obtained in the synthesis) in the sample and the second is related to the complete degradation of the polymeric chains. The residue that remained (4.1%) is the ash from the decomposition of the copolymer.

The first mass loss in the Poly(Sty-co-DVB), Poly(Sty-co-DVB)/Magnetite and Poly(Sty-co-DVB)/Magnetite/Oleic Acid samples was very small: 1.9%, 3.1% and 2.4%, respectively, occurring between temperatures of 30 °C and 300 °C. The second mass loss, within the temperature range of 300 °C to 600 °C, was 94.0% for the Poly(Sty-co-DVB) sample, 88.2% for the Poly(Sty-co-DVB)/Magnetite sample and 87.5% for the Poly(Sty-co-DVB)/Magnetite sample /Oleic acid. The degradation maximum temperatures of these two losses were, respectively, 135±2 °C and 411±2 °C. The first loss, as mentioned above, may be due to the presence of traces of solvents (washing water and/or hydrochloric acid and absolute ethanol used to neutralize the product obtained in the synthesis) in the sample. This result corroborates the results observed and reported from the analysis of FTIR spectra for all samples. The samples of the Poly(Sty-co-DVB) polymer with magnetite and with magnetite and oleic acid showed a slightly greater mass loss than the one presented by the sample with the polymer alone. The presence of magnetite may have influenced this small difference. The second mass loss is related to the complete degradation of the chain's polymers of Poly(Sty-co-DVB). The only significant difference between these curves was the percentage of residue left over, since in the Poly(Sty-co-DVB)/Magnetite and Poly(Sty-co-DVB)/Magnetite/Oleic Acid samples, in addition to the ash of the organic matter, there is magnetite that does not undergo decomposition in the analyzed temperature range. Considering that the percentage of ash is 4.1% (residue obtained by TGA curve for the Poly(Sty-co-DVB) sample – Table 3), it can be estimated that the percentage of magnetite present in the Poly(Sty-co-DVB)/Magnetite and Poly(Sty-co-DVB)/Magnetite/Oleic Acid samples is, respectively, 4.6 and 6.0%. These values are below the amount added in the synthesis of these systems (10% m/m), indicating that there was a loss of magnetite in their synthesis procedure. In the TGA curve for the sample Poly(Sty-co-DVB)/Magnetite/Oleic Acid, the loss of mass relative to oleic acid was not detected. Perhaps it was extracted or lost during some synthesis step of this sample. The percentage of magnetite present in the Poly(Sty-co-DVB)/Magnetite with Oleic Acid sample was slightly higher than that present in the sample without oleic acid. This acid probably influenced a greater incorporation of magnetite in the polymer during the synthesis, even though it was not present in the characterization of the synthesized sample.

The syntheses of the polymer and copolymers based on styrene-divinylbenzene with the modification by addition of magnetite and magnetite with oleic acid coating were successful, conform determinate from the three techniques used for characterizations these systems.

## Declaration of Competing Interest

The authors declare that they have no known competing financial interests or personal relationships that could have appeared to influence the work reported in this paper.
